# Clinical Utility of Adapted Modified Canine Activity Index (aMCAI) in Canine Acute Pancreatitis: A Prospective Observational Study

**DOI:** 10.3390/ani16091292

**Published:** 2026-04-22

**Authors:** Veerada Wachirodom, Sathidpak N. Assawarachan, Suwicha Kasemsuwan, Melanee Suksamranthaweerat, Kasamapohn Hutachinda, Monchanok Vijarnsorn, Narudee Kashemsant

**Affiliations:** 1 Endocrinology and Gastroenterology Unit, Kasetsart University Veterinary Teaching Hospital, Faculty of Veterinary Medicine, Kasetsart University, 50 Ngamwongwan Rd., Lat Yao, Jatujak, Bangkok 10900, Thailand; veerada.w@ku.th (V.W.); sathidpak.n@ku.ac.th (S.N.A.); 2 Department of Companion Animal Clinical Sciences, Faculty of Veterinary Medicine, Kasetsart University, 50 Ngamwongwan Rd., Lat Yao, Jatujak, Bangkok 10900, Thailand; monchanok.vi@ku.th; 3 Department of Veterinary Public Health, Faculty of Veterinary Medicine, Kasetsart University, Kamphaeng Saen Campus, Nakhon Pathom 73140, Thailand; fvetswk@ku.ac.th; 4 Internal Medicine Unit, Kasetsart University Veterinary Teaching Hospital, Faculty of Veterinary Medicine, Kasetsart University, 50 Ngamwongwan Rd., Lat Yao, Jatujak, Bangkok 10900, Thailand; melanee.su@ku.th; 5 Cardiology Unit, Kasetsart University Veterinary Teaching Hospital, Faculty of Veterinary Medicine, Kasetsart University, 50 Ngamwongwan Rd., Lat Yao, Jatujak, Bangkok 10900, Thailand; kasamaporn.h@ku.th; 6 Department of Physiology, Faculty of Veterinary Medicine, Kasetsart University, 50 Ngamwongwan Rd., Lat Yao, Jatujak, Bangkok 10900, Thailand

**Keywords:** canine acute pancreatitis, Modified Canine Activity Index, clinical severity score, prognostic index

## Abstract

**Simple Summary:**

Acute pancreatitis is a common and potentially life-threatening condition in dogs. Early recognition of clinical severity is important for guiding treatment decisions. Most existing prognostic scoring systems are either inconsistent, complex, or require laboratory data. Among those scoring systems, the Modified Canine Activity Index (MCAI) is particularly interesting because it relies solely on clinical observations and shows potential prognostic value. However, its original fecal scoring relies on subjective descriptive definitions, which may introduce inter-observer variability. Therefore, this study evaluated an adaptation called the Adapted Modified Canine Activity Index (aMCAI), which was based on the original version. This study evaluated aMCAI scores in dogs with acute pancreatitis to investigate whether the aMCAI could provide prognostic information and monitor clinical progression using only clinical observations. The findings indicated that lower scores on Day 5 were associated with survival, suggesting that this scoring system may help in monitoring clinical improvement and provide supportive information for clinical decision-making.

**Abstract:**

Assessing disease severity and prognosis in canine acute pancreatitis (AP) remains a major clinical challenge. This study evaluated the clinical utility of the Adapted Modified Canine Activity Index (aMCAI), a clinical scoring system refined from the original MCAI. A prospective observational study was conducted on 42 dogs diagnosed with AP, with aMCAI scores assessed on Days 1, 3, and 5. A linear mixed model (LMM) was used to analyze score progression over time and differences between survivors and non-survivors. Receiver operating characteristic (ROC) curves evaluated the prognostic accuracy for 30 day survival. The LMM analysis revealed that non-survivors had significantly higher aMCAI scores than survivors (*p* = 0.035), and overall scores decreased significantly over time (*p* < 0.001). ROC analysis showed poor discrimination on Days 1 and 3. However, on Day 5 the aMCAI demonstrated good prognostic performance (AUC = 0.813, *p* < 0.001). A cutoff value of ≥2.5 on Day 5 yielded 100% sensitivity, a negative likelihood ratio of 0.00 and a 100% negative predictive value, providing clinically relevant prognostic information. These findings suggest that the aMCAI is a practical tool for monitoring disease progression and may support the identification of dogs with a high likelihood of survival.

## 1. Introduction

Acute pancreatitis (AP) is the most common pancreatic disease in dogs and presents with a wide spectrum of clinical signs, ranging from mild disease to severe systemic involvement and multiple organ failure [1,2]. Poor outcomes are often associated with delayed or inadequate treatment, partly due to the absence of reliable early indicators of disease severity. In human medicine, early identification of severe AP is considered essential for initiating appropriate therapy to reduce mortality [3]. In contrast, severity assessment of canine AP remains challenging, with reported mortality rates remaining high, in the range of 27–58% [1,4,5,6]. Therefore, it is essential to develop practical and reliable tools for early severity assessment in dogs to inform treatment decisions and improve patient outcomes [7,8].

Several scoring systems have been proposed to evaluate disease severity and predict outcomes in canine AP. However, each has notable limitations. The Organ Score (OS), introduced in 1998, incorporates five organ-related parameters and biochemical data. However, it relies on diagnostic criteria for canine AP (elevated amylase and lipase) that are no longer considered accurate [9]. The Clinical Severity Index (CSI), developed in 2008, was based on an initial evaluation of nine systems and found that cardiovascular, respiratory, gastrointestinal integrity, and blood pressure parameters were associated with mortality [5]. However, subsequent studies reported inconsistent results and poor correlations with clinical outcomes [10,11]. More recently, the Canine Acute Pancreatitis Severity (CAPS) score, which included SIRS status, coagulation disorders, increased creatinine levels, and hypocalcemia, demonstrated high sensitivity and specificity in one validation cohort [1]. However, while one study identified a CAPS score ≥ 11 as being significantly associated with clinical outcomes [12], subsequent studies did not support this threshold. One study reported no significant difference [11] and another reported that dogs with a CAPS score ≥ 11 were not associated with non-survival [13]. In addition, the Acute Patient Physiologic and Laboratory Evaluation (APPLE_full_) score system, originally developed for critically ill dogs rather than specifically for acute pancreatitis, has been evaluated regarding canine AP. For example, a 2025 study reported that the APPLE_full_ score calculated from the first available data was significantly different between survivors and non-survivors and demonstrated discriminatory ability for predicting prolonged hospitalization. However, high APPLE_full_ scores should be interpreted with caution due to the low case mortality rate in that study [11].

The Modified Canine Activity Index (MCAI) was originally adapted from the Canine Inflammatory Bowel Disease Activity Index (CIBDAI); however, unlike CIBDAI, the MCAI includes seven parameters specific to acute pancreatitis: activity, appetite, vomiting, cranial abdominal pain, dehydration status, fecal consistency, and the presence of blood in the stool. An initial study found significant differences in the MCAI scores between the survivors and non-survivors, suggesting that the MCAI may be useful for prognosis and the monitoring treatment in canine AP [10]. However, that study was limited by its small sample size (*n* = 13). Subsequently, the MCAI was applied to evaluate the efficacy of fuzapladip sodium for the treatment of AP in dogs [14], although disease severity assessment was not the primary focus. More recently, a large-scale study (*n* = 494) demonstrated a significant difference in MCAI scores between survivors and non-survivors and showed predictive ability for hospitalization duration. However, the low mortality rate and low positive predictive value (PPV) for death led the authors to caution against using high MCAI scores to predict mortality [11]. Additionally, the retrospective design may have introduced limitations related to data completeness and potential confounding factors. A key limitation of the original MCAI relies on subjective descriptive criteria for fecal consistency scoring and the definition of blood in the stool, which may lead to inter-observer variability and reduce reproducibility in clinical practice. To address these limitations and fill the gap in prospective evidence, the present study aimed to develop the Adapted Modified Canine Activity Index (aMCAI) and evaluate it in a prospective observational setting. The detailed scoring criteria and component definitions of the aMCAI are described in Section 2. The aMCAI incorporates the standardized Purina Fecal Scoring System to replace the original subjective fecal consistency descriptors and explicitly defines blood in the stool as the presence of either visible blood or melena, thereby reducing interpretive ambiguity. As the aMCAI relies exclusively on clinical observations, it is practical and applicable in routine clinical settings. We hypothesize that the aMCAI score may provide prognostic information and facilitate monitoring of clinical progression in canine AP.

## 2. Materials and Methods

### 2.1. Study Population and Inclusion Criteria

A prospective study was undertaken based on dogs diagnosed with AP at the Kasetsart University Veterinary Teaching Hospital in Bangkok, Thailand, from April 2022 to April 2023. The owners of all the participating animals provided informed consent. The study was approved by the Kasetsart University Institutional Animal Care and Use Committee, Kasetsart University, Bangkok, Thailand (ACKU/65-VET-006).

Dogs were eligible to be enrolled if they presented with at least two clinical signs associated with AP (vomiting, diarrhea, anorexia, abdominal pain) for fewer than seven days; had at least two sonographic features of AP based on abdominal ultrasound findings within 48 h of presentation, including pancreatic enlargement, hypoechoic parenchyma, hyperechoic surrounding mesenteric fat, and peripancreatic fluid [15,16]; and had an abnormal SNAP canine pancreatic lipase (cPL) test result (IDEXX Laboratories, Inc.; Westbrook, ME, USA) on Day 1 (at presentation). These criteria ensured that each dog met the published guidelines for clinically probable AP [17].

Dogs with two or more previous episodes of AP were excluded, as recurrent episodes may indicate underlying chronic pancreatitis rather than true acute disease [18]. Dogs younger than one year or weighing less than 2 kg were also excluded. Dogs with severe anemia (hematocrit <20%) at presentation or developing during the study were excluded, as severe anemia can independently cause clinical signs such as lethargy and weakness that overlap with components of the aMCAI score, leading to an overestimation of AP-related disease severity. Additionally, dogs were not included in the study if they had a concurrent disease in which AP was considered a minor disease process (immune-mediated hemolytic anemia, pyometra, or gall bladder rupture).

### 2.2. Data Collection and Clinical Assessments

For each enrolled dog, demographic and clinical data were collected prospectively during the study. This included age, sex, breed, sterilization history, and body condition score (BCS); furthermore, clinical signs and physical examination findings were recorded at presentation.

An adapted clinical scoring system, termed the Adapted Modified Canine Activity Index (aMCAI), was developed based on the original MCAI. The aMCAI comprises seven parameters: activity, appetite, vomiting, cranial abdominal pain, dehydration status, fecal consistency, and the presence of blood in the stool. Five parameters (activity, appetite, vomiting, cranial abdominal pain, and dehydration status) were retained unchanged from the original MCAI, while two parameters were modified as follows: (1) fecal consistency was refined using the standardized 7-point Purina Fecal Scoring system and grouped into four categories; and (2) blood in the stool was defined as the presence of either visible blood or melena. The total aMCAI score was calculated as the sum of all parameter scores, ranging from 0 to 19. Detailed scoring criteria and parameter definitions are provided in Table 1, and the correspondence between the aMCAI and MCAI fecal consistency scores are shown in Table 2. The aMCAI scores were assessed and recorded on Days 1, 3, and 5.

The dogs were divided into two groups: survivors and non-survivors. Survivors were defined as dogs that survived for more than 30 days after presentation. Conversely, non-survivors consisted of dogs that died within 30 days of presentation.

### 2.3. Statistical Analysis

Descriptive statistics were used to summarize the baseline characteristics, with continuous variables being tested for normality using the Shapiro–Wilk test and presented as mean ± standard deviation (SD) values or as median values with an interquartile range (IQR) as appropriate. Categorical data were expressed as a counts and percentages (%). Comparisons between survivor and non-survivor dogs were performed using the Mann–Whitney U test for continuous variables and Fisher’s exact test for categorical variables.

Changes in the aMCAI scores over time (Days 1, 3, and 5) and the comparison between the survival and non-survival groups were analyzed using a linear mixed model (LMM) with restricted maximum likelihood estimation. The model applied Group, Day, and their interaction (Group × Day) as fixed effects, while the individual dog identifier was specified as a random intercept to account for between-subject variability. An unstructured covariance structure was used to model residual dependencies across time points. Significance was assessed using Type III F-tests, with post hoc Bonferroni-adjusted estimated marginal means (EM means) being used for pairwise comparisons. Model assumptions were verified using residual diagnostics. The normality of residuals was assessed using the Shapiro–Wilk test, and homoscedasticity was evaluated based on plots of residuals versus predicted values.

Receiver operating characteristic (ROC) curves were generated to evaluate the ability of the aMCAI to predict survival at each time point. The following parameters were calculated: the area under the curve (AUC), sensitivity, specificity, positive likelihood ratio (LR+), negative likelihood ratio (LR−), PPV and negative predictive value (NPV). Optimal cutoff values were selected based on the Youden index.

For all analyses, a *p*-value *<* 0.05 was considered statistically significant. All statistical analyses were performed using the IBM SPSS Statistics software (version 31; IBM Corp., Armonk, NY, USA). Figures were generated using Python (version 3.12) with Seaborn and Matplotlib libraries (Python Software Foundation, Wilmington, DE, USA).

## 3. Results

In total, 45 dogs were enrolled initially. Of these, 42 dogs met the inclusion criteria for data analysis. The other three dogs were excluded due to deaths from causes unrelated to AP. The study population consisted of 17 males (12 neutered, 5 intact), and 25 females (23 spayed, 2 intact). The median age was 11 years (IQR 9–12). The dogs represented a variety of breeds, comprising mixed-breed dogs (*n* = 9; 21.43%), Chihuahuas (*n* = 8), Pomeranians (*n* = 6), Poodles (*n* = 6), Shih Tzus (*n* = 3), French Bulldogs (*n* = 2), Beagles (*n* = 2), and one (2.38%) each of Dachshund, German Shepherd, Golden Retriever, Thai Bang Kaew, Jack Russell Terrier, and Welsh Corgi. Among the dogs, 66.7% (*n* = 28) were classified as overweight, with a BCS of 6/9 or higher. There were no significant differences in age (*p =* 0.117), sex (*p* = 0.477), sterilization status (*p* = 0.353), and overweight condition (*p* = 1.0) between survivors and non-survivors (Table 3).

Based on 30 day survival following diagnosis, 31 dogs were classified as survivors and 11 as non-survivors, corresponding to a mortality rate of 26.19%.

**Figure 1:**
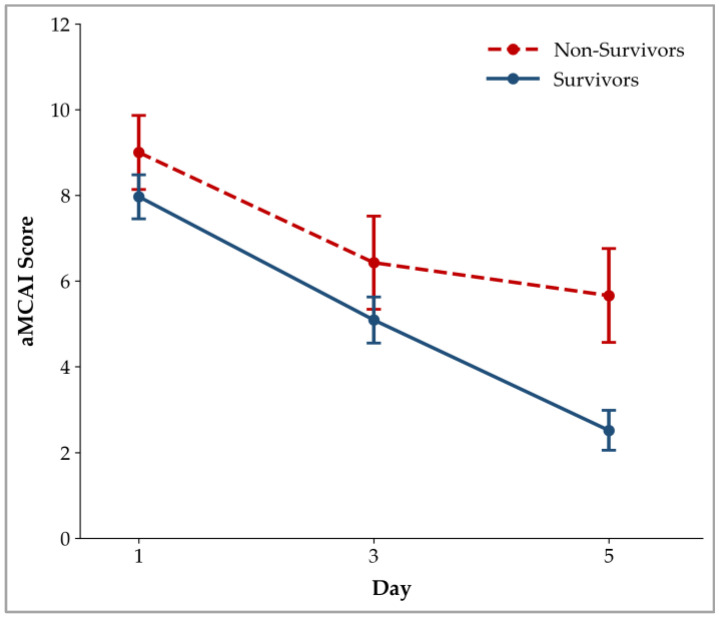
Estimated marginal means of aMCAI scores in survivor and non-survivor dogs on Days 1, 3, and 5. Data presented as mean ± SE values.

**Figure 2:**
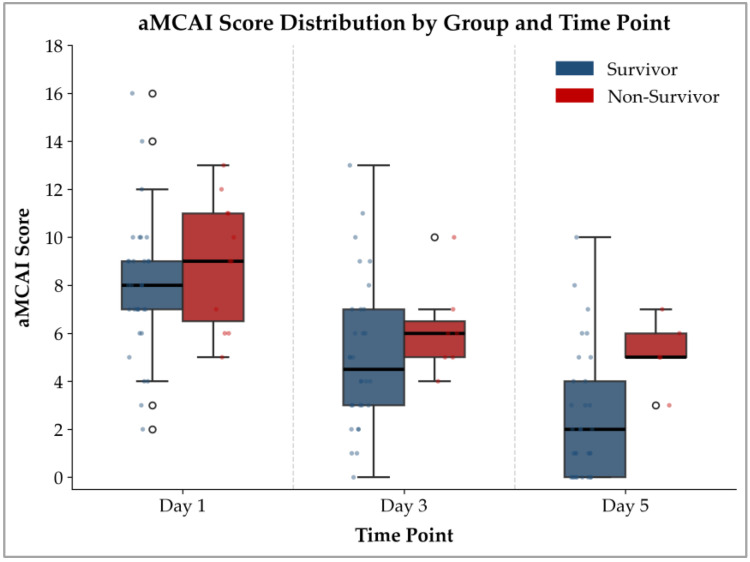
Distribution of aMCAI scores in survivor and non-survivor groups at Days 1, 3, and 5. Dots represent individual data points, and open circles indicate outliers.

The LMM revealed no significant interaction between Group and Day (*p* = 0.249), indicating that the pattern of change in aMCAI scores over time did not differ significantly between the survivor and non-survivor groups. However, a significant main effect of Group was observed (*p* = 0.035), with non-survivors having significantly higher aMCAI scores than survivors. In addition, there was a significant main effect of Day *(p* < 0.001), demonstrating that the aMCAI scores changed significantly across the three assessment time points (Days 1, 3, and 5) in the overall cohort. Modeling assumptions were met, with normally distributed residuals and no evidence of heteroscedasticity. The estimated marginal means of the aMCAI scores derived from the LMM are presented in Table 4 and illustrated in Figure 1. The distribution of aMCAI scores across time points and groups is further visualized in Figure 2, which demonstrates a progressive decline in scores over time in both groups, with non-survivors consistently showing higher scores than survivors throughout the study period.

**Figure 3:**
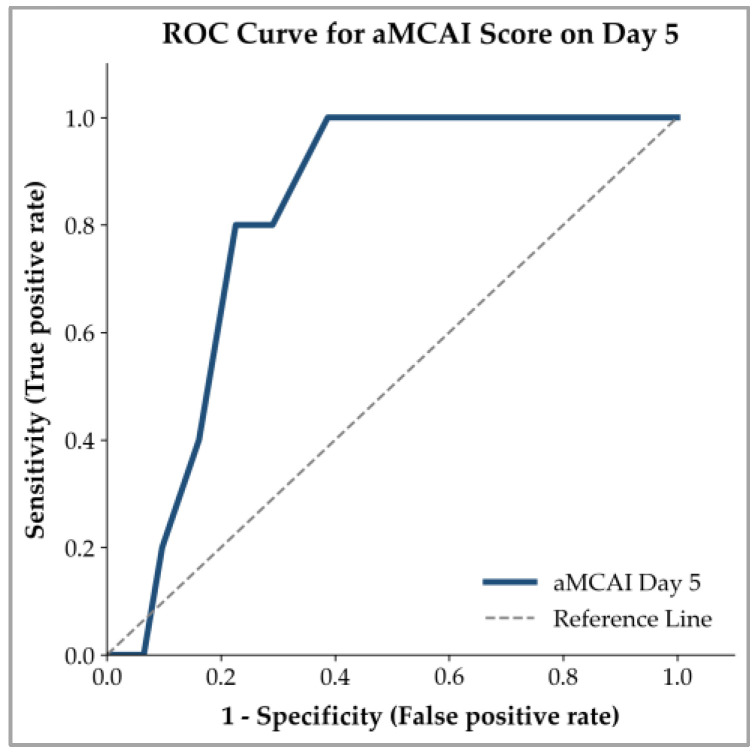
Receiver operating characteristic (ROC) curve of the aMCAI score on Day 5 for predicting 30 day survivors.

ROC curve analysis was performed to evaluate the prognostic efficacy of the aMCAI score on Days 1, 3, and 5. On Day 1, the aMCAI score failed to demonstrate significant discriminatory power between survivors and non-survivors (AUC = 0.611, 95% CI: 0.403–0.820, *p* = 0.294), with no clinical cutoff value being identified. The prognostic performance showed a slight improvement on Day 3, but this was not significant (AUC = 0.643, 95% CI: 0.464–0.821, *p* = 0.117). In contrast, the aMCAI score on Day 5 demonstrated good significant prognostic ability (AUC = 0.813, 95% CI: 0.672–0.954, *p* < 0.001). The ROC curve for Day 5 is presented in Figure 3. The optimal cutoff value was ≥2.5, which resulted in a sensitivity of 100% and a specificity of 61.3%. In addition, this threshold had an LR+ of 2.58 and an LR− of 0.00. Furthermore, this cutoff yielded a PPV of 47.83% and an NPV of 100%, based on the observed mortality rate of the cohort.

In an exploratory comparison, we applied the concept from a previous study [14] in which removing the fecal consistency and blood in stool items from the MCAI to create the MCAI5 improved the statistical significance of score changes between the treatment and placebo groups [14]. The MCAI5 model showed significant effects for Group and Day in the LMM (Group *p* < 0.001; Day *p* < 0.001), with no significant interaction (*p* = 0.117). The ROC analysis showed that the MCAI5 had significant prognostic ability, with moderate performance on Day 3 (AUC 0.762, *p* = 0.001) and further improvement by Day 5 (AUC 0.877, *p* < 0.001). At the Day 5 cutoff of ≥2.5, sensitivity was 100% and specificity was 67.7%, while the LR+, LR−, PPV, and NPV were 3.10, 0.00, 52.35% and 100%, respectively. A summary of the prognostic performance for both scores is presented in Table 5.

## 4. Discussion

The original MCAI was selected as the foundation for this study due to its practicality and reliance on commonly observed clinical signs in canine AP. A preliminary study in 2021 suggested a correlation between the MCAI and clinical outcomes, although the limited sample size (*n* = 13) precluded firm conclusions [14]. A subsequent larger retrospective study (*n* = 494) demonstrated that the MCAI could differentiate between survivors and non-survivors at the first available clinical assessment. However, the low mortality rate resulted in a low PPV (11.4%), indicating that the MCAI should be interpreted cautiously when used for mortality prediction [11].

Furthermore, the fecal consistency criteria of the original MCAI relied on descriptive terminology without a standardized scoring system, which may have reduced objectivity and consistency. To address this limitation, the current study monitored dogs prospectively on Days 1, 3, and 5 using an adapted version (the aMCAI), which incorporated the 7-point Purina Fecal Scoring system and defined blood in the stool to include both visible blood and melena. We hypothesized that these refinements would enhance scoring precision and improve the utility of the aMCAI for predicting prognosis and monitoring treatment response.

The LMM analysis showed that aMCAI scores differed significantly between survivors and non-survivors, with non-survivors consistently having higher scores, indicating that the aMCAI had prognostic potential. Although both groups had score reductions over time, the absence of a significant interaction effect indicated that the pattern of score reduction was comparable. Therefore, evaluating the score using a prognostic cutoff at a specified time point was clinically useful, whereas the downward trend in scores alone could not be used to predict outcomes.

Regarding prognostic accuracy, the ROC curve analysis revealed limited discriminatory performance on Days 1 and 3. This likely reflected the early pathophysiology of AP, where the disease progression involves multiple interacting inflammatory pathways, including enzyme activation, cytokine release, and microcirculatory disturbances [2,6,20]. During this phase, the balance between protective mechanisms and injurious processes has not yet fully determined disease severity, and cumulative stressors may not yet exceed the host’s compensatory capacity [20], which may in part explain why clinical presentations between survivors and non-survivors overlap substantially at these early time points.

On Day 5, the aMCAI showed good prognostic capability. The improved prognostic performance observed at this time point may reflect disease progression, characterized by the amplification of inflammatory responses and the development of tissue injury [2]. At this stage, differences in disease severity become more clinically apparent, leading to a clearer separation between survivors and non-survivors. Our findings are consistent with a previous study reporting a low PPV for mortality prediction using the original MCAI at initial assessment, despite a statistically significant difference between survivors and non-survivors [11]. Although direct comparison is limited by differences in scoring systems and study design, the aMCAI similarly demonstrated limited prognostic ability at earlier time points in our study, with strong performance on Day 5. This further supports its utility as a tool for monitoring clinical improvement rather than for initial prognostication.

The clinical value of the aMCAI lies in its serial monitoring, particularly the strong prognostic performance on Day 5. Using a cutoff of ≥2.5 yielded 100% sensitivity, an LR− of 0.00, and a 100% NPV, indicating that the aMCAI on Day 5 may serve as a screening tool for ruling out mortality in dogs with AP. Notably, the combination of high sensitivity and moderate specificity (61.3%) indicates that a proportion of surviving dogs also had scores ≥2.5. This is consistent with the observed distribution of scores (Figure 3), where overlap between survivor and non-survivor groups was present, particularly at higher score ranges. Therefore, while a score of 0–2 is strongly associated with survival, a higher score (≥3) does not necessarily indicate death but rather reflects an increased risk of poor outcomes. Accordingly, the aMCAI on Day 5 should be interpreted as a tool for risk stratification rather than a definitive determinant of clinical decisions. Low scores may support a favorable prognosis, whereas higher scores warrant closer monitoring and continued clinical evaluation. Importantly, the aMCAI should not be used as a standalone criterion for clinical decision-making, such as discharge, but rather as part of a comprehensive clinical assessment.

To further investigate the factors influencing prognostic timing, we conducted an exploratory analysis of the MCAI5 based on concepts from a previous study [14]. Notably, the MCAI5 showed earlier discriminatory ability on Day 3, unlike the aMCAI, suggesting that diarrhea may introduce confounding factors, as it may be a manifestation of AP, pre-existing conditions, or other concurrent gastrointestinal diseases [21,22,23]. However, diarrhea is the second-most common clinical sign in canine AP [23]. Excluding the assessment of fecal consistency and blood in the stool prevents monitoring of these symptoms and associated complications. Consequently, disregarding these signs may risk overlooking ongoing gastrointestinal injury, even if other clinical signs improve. Recent evidence has further highlighted that gastrointestinal involvement is a clinically relevant component of acute pancreatitis and is associated with disease severity, systemic inflammatory responses, and poorer clinical outcomes [24,25]. In this context, parameters reflecting gastrointestinal status, such as fecal-related findings, may provide meaningful information when interpreted as part of a composite clinical index and assessed serially over time.

Consistent with this, on Day 5, both scoring systems performed comparably, each achieving values of 100% sensitivity, LR− 0.00 and 100% NPV. Although the MCAI5 had slightly higher specificity, LR+ and PPV, the differences were small and unlikely to be clinically meaningful. While the MCAI5 offers simplicity and earlier prediction, the aMCAI may provide a more comprehensive evaluation of gastrointestinal recovery which may help clinicians track disease progression and response to therapy, making it particularly valuable for clinical monitoring rather than just for early prognostication. Additionally, the MCAI5 comparison was exploratory rather than pre-specified; therefore, these findings should be interpreted with caution.

Furthermore, it was notable that PPV and NPV were influenced by the mortality prevalence in our population (26.19%) and might vary in other clinical settings. In contrast, likelihood ratios (LR+ and LR−) represent intrinsic measures of test performance and are independent of disease prevalence. Therefore, they provide a more stable estimate of prognostic performance across different populations. The choice of cutoff value affected these performance metrics. While the current study utilized the optimal Youden index, using alternative cutoff values would result in different predictive outcomes.

In summary, this study suggests that the aMCAI is more useful for monitoring clinical response than for early prognostication at initial presentation. Although serial assessment captures a downward trend in scores over time, this apparent improvement alone is not a reliable indicator of outcome. Notably, scores also decreased in non-survivors but failed to reach a recovery threshold. Therefore, the prognostic value of the aMCAI is inherently dependent on both time and score. A favorable prognosis is generally associated with not only clinical improvement but also the attainment of a defined recovery threshold, particularly a score below 2.5 by Day 5. These findings emphasize that prognostic interpretation should be based on whether sufficient clinical recovery is achieved within an appropriate timeframe, rather than on declining trends alone.

The present study has several limitations. First, the study population was relatively small, particularly in the non-survivor group, which may have affected the precision of analyses and limited the statistical power; consequently, the perfect sensitivity and NPV observed on Day 5 should be interpreted as preliminary estimates pending validation in larger cohorts. Second, as a single-center study, the findings may not be fully generalized to other clinical settings with different patient distributions or management protocols. Third, the aMCAI assessments were performed only on Days 1, 3, and 5, potentially missing day-to-day fluctuations in clinical signs. Lastly, although all dogs received standard-of-care treatment including intravenous fluid therapy, analgesia, antiemetics, gastric acid suppression, and nutritional support, specific drug selections and dosages were determined based on individual clinician judgment and each patient’s clinical needs, rather than a strictly standardized protocol. This variability in treatment may have introduced confounding factors that could influence the observed outcomes.

## 5. Conclusions

The aMCAI on Day 5 can differentiate between survivors and non-survivors in dogs with AP, with scores below 2.5 associated with a high likelihood of survival in this cohort. As it relies solely on clinical assessment, the aMCAI offers a practical and accessible tool for clinicians, particularly in settings where repeated laboratory testing may be limited. While serial assessments provide useful information on changes in clinical status, prognostic evaluation is most informative when performed at an appropriate time point using a defined cutoff. Importantly, the aMCAI should not be used as a standalone criterion for overall clinical decision-making. Given the limited sample size, potential for interobserver variability, and the preliminary nature of perfect sensitivity and NPV estimates, further prospective, multicenter studies are warranted to validate its prognostic performance and clinical utility.

## Figures and Tables

**Table 1:** Scoring system of the adapted Modified Canine Activity Index (aMCAI).

Parameter	Score	Description	Score Range
Activity	0	Normal (as usual)	0–3
	1	Slightly decrease (the animal stands less than usual)	
	2	Moderate decreased (the animal reluctant stand up)	
	3	Severely decreased (the animal cannot stand up)	
Appetite (voluntary food intake)	0	Normal (the animal ate more than 75% of offered food)	0–3
	1	Slightly decrease (the animal ate about 50% of offered food)	
	2	Moderate decreased (the animal ate about 25% of offered food)	
	3	Severely decreased (the animal ate less than 25% or refused food)	
Vomiting	0	None	0–3
	1	1–2 times/day	
	2	3–4 times/day	
	3	≥5 times/day	
Cranial abdominal pain	0	None (no signs of pain)	0–3
	1	Mild (abdominal wall resistance or other signs of pain are elicited upon palpation of abdomen, the animal moves slowly or is less responsive)	
	2	Moderate (the animal wall resists palpation, is reluctant to move when encouraged, does not lie on its side)	
	3	Severe (persistent vocalization, howling, and/or insomnia)	
Dehydration	0	None (<5%, no signs of dehydration)	0–3
	1	Mild (5%, slight loss of skin elasticity)	
	2	Moderate (6–8%, decreased skin turgor, slight delayed capillary refill time, dry mucous membranes, sunken eyes)	
	3	Severe (≥10%, severely decreased skin turgor, delayed capillary refill time, deeply sunken eyes, severely dry mucous membranes)	
Fecal consistency	0	Purina fecal score 2–3	0–3
	1	Purina fecal score 4–5	
	2	Purina fecal score 6	
	3	Purina fecal score 7	
Blood in stool/melena	0	Absent	0–2
	1	Present (visible blood or melena)	
Total aMCAI score		Sum of all parameters	0–19

**Table 2:** Comparison of fecal consistency scoring between Adapted Modified Canine Activity Index (aMCAI) and MCAI.

aMCAI Score	Corresponding Purina Fecal Score	Fecal Characteristics *	MCAI Score
0	2–3	Score 2: Firm, but not hard; pliable; segmented appearance; leaves little or no surface residue when picked up.	0 = Normal (well formed)
		Score 3: Log shape; moist surface; little or no visible segmentation; leaves surface residue but holds form when picked up.	
1	4–5	Score 4: Very moist and soggy; log shaped; leaves surface residue and loses form when picked up.	1 = Soft (slightly watery and poorly formed)
		Score 5: Very moist but has a distinct shape; present in piles rather than logs; leaves surface residue and lose form when picked up.	
2	6	Has texture but no defined shape; present as piles or spots; leaves surface residue when picked up.	2 = Diarrhea (No form and runny)
3	7	Watery; no texture; present in flat puddles.	3 = Watery (watery with no solids and pale)

* Fecal characteristics adapted from the Purina Fecal Scoring System [19].

**Table 3:** Demographic and baseline characteristics of dogs with acute pancreatitis.

Variable	Overall (*n* = 42)	Survivor (*n* = 31)	Non-Survivor (*n* = 11)	*p*-Value
Age (years), Median [IQR]	11 [10,11,12,13]	11 [10,11,12,13]	13 [10,11,12,13,14,15]	0.117 ^1^
Sex (male/female)	17/25	14/17	3/8	0.477 ^2^
Sterilization status (sterilized/intact)	35/7	27/4	8/3	0.353 ^2^
Overweight (BCS ≥ 6/9, n (%))	28 (66.7%)	21 (67.7%)	7 (63.7%)	1.000 ^2^

^1^ Ages compared using the Mann–Whitney U test. ^2^ Categorical variables compared using Fisher’s exact test.

**Table 4:** Estimated means aMCAI scores in survivors and non-survivors over time (linear mixed model estimate).

Day	Group	Mean (LMM Estimate)	SE	95% CI
Day 1	Non-Survivors	9.000	0.862	7.258–10.742
	Survivors	7.968	0.514	6.930–9.006
Day 3	Non-Survivors	6.428	1.087	4.229–8.628
	Survivors	5.091	0.536	4.005–6.177
Day 5	Non-Survivors	5.661	1.095	3.446–7.876
	Survivors	2.516	0.465	1.573–3.460

**Table 5:** Prognostic efficacy of aMCAI and MCAI5 score for 30 day survival.

aMCAI									
Day	AUC (95%CI)	*p*-Value	Optimal Cut-Off	Sensitivity (%)	Specificity (%)	LR+	LR−	PPV (%)	NPV (%)
Day 1	0.611 (0.403–0.820)	0.294	-	-	-			-	-
Day 3	0.643 (0.464–0.821)	0.117	-	-	-			-	-
Day 5	0.813 (0.672–0.954)	<0.001 *	≥2.5	100.0	61.3	2.58	0.00	47.83	100.0
**MCAI5**									
**Day**	**AUC (95%CI)**	***p*-Value**	**Optimal Cut-Off**	**Sensitivity (%)**	**Specificity (%)**	**LR+**	**LR−**	**PPV (%)**	**NPV (%)**
Day 1	0.626 (0.442–0.811)	0.180	-	-	-			-	-
Day 3	0.762 (0.602–0.922)	0.001 *	≥3.5	100.0	46.7	1.88	0.00	39.97	100.0
Day 5	0.877 (0.759–0.996)	<0.001 *	≥2.5	100.0	67.7	3.10	0.00	52.35	100.0

* Significantly different (*p* < 0.05).

## Data Availability

The data presented in this study are included within the article. The raw data supporting the findings of this study are available from the corresponding author upon reasonable request.

## Abbreviations

AP	Acute pancreatitis
MCAI	Modified Canine Activity Index
aMCAI	Adapted Modified Canine Activity Index
CSI	Clinical Severity Index
CAPS	Canine Acute Pancreatitis Severity
APPLE_full_	Acute Patient Physiologic and Laboratory Evaluation score system
CIBDAI	Canine Inflammatory Bowel Disease Activity Index
BCS	Body condition score
NPV	Negative predictive value
PPV	Positive predictive value
LMM	Linear mixed model
IQR	Median and interquartile range
EM Means	Estimated marginal means
AUC	Area under the curve
ROC	Receiver operating characteristic
